# Efficacy and safety of AAV RPGR gene therapy in X-linked retinitis pigmentosa: a systematic review and meta-analysis

**DOI:** 10.1186/s12967-026-08709-7

**Published:** 2026-08-10

**Authors:** Kai-Yang Chen, Hoi-Chun Chan, Chi-Ming Chan

**Affiliations:** 1 Department of General Medicine, Chang Gung Memorial Hospital (Linkou branch), Taoyuan, Taiwan; 2 School of Pharmacy, China Medical University, Taichung, Taiwan; 3 Department of Ophthalmology, Cardinal Tien Hospital, New Taipei City, Taiwan; 4 School of Medicine, Fu Jen Catholic University, New Taipei City, Taiwan

**Keywords:** X-linked retinitis pigmentosa, Adeno-associated viral vectors, Gene therapy, Retinitis pigmentosa GTPase regulator

## Abstract

**Background:**

X-linked retinitis pigmentosa (XLRP) represents a severe inherited retinal dystrophy associated with pathogenic variants in the retinitis pigmentosa GTPase regulator (RPGR) gene. Adeno-associated virus (AAV)-mediated RPGR gene augmentation is designed to preserve photoreceptor structure and function.

**Objectives:**

The purpose of this study was to critically appraise and quantitatively synthesize the efficacy and safety evidence for AAV-RPGR gene therapy in X-linked retinitis pigmentosa.

**Methods:**

Scopus, PubMed, the Cochrane Library, ScienceDirect, and Google Scholar were searched from inception through July 11, 2026. Two reviewers independently screened records, two reviewers assessed risk of bias, and extracted data were verified by a second reviewer. Proportions were synthesized using inverse-variance fixed-effect logit models with a 0.5 continuity correction for zero or all-event cells; DerSimonian-Laird random-effects models were used as sensitivity analyses. Cohort linkage, dose-stratified safety, and overlap-adjusted analyses were performed.

**Results:**

The search identified 571 records and included 12 clinical reports. Pooled retinal sensitivity improvement was 73.8% (95% confidence interval, 56.0%-86.1%; 25/33 participants), and pooled visual function improvement was 52.3% (95% confidence interval, 38.0%-66.2%; 28/52 participants). The pooled adverse-event proportion was 42.6% (95% confidence interval, 27.2%-59.5%; 42/90 participants), intraocular inflammation was 45.5% (95% confidence interval, 34.6%-56.8%; 36/81 participants), and intraocular-pressure elevation was 34.9% (95% confidence interval, 24.2%-47.4%; 22/63 participants). Product-specific dose analyses showed greater inflammatory or ocular serious adverse-event frequencies at higher vector exposure.

**Conclusion:**

AAV-RPGR gene therapy demonstrates clinically relevant functional signals across multiple outcome domains with a structured and monitorable ocular safety profile. Cohort-linked synthesis, dose-specific interpretation, standardized outcome definitions, and long-term multinational follow-up provide a rigorous framework for subsequent clinical development.

## Introduction

Retinitis pigmentosa encompasses a heterogeneous group of inherited retinal dystrophies characterized by the progressive degeneration of photoreceptors, resulting in irreversible vision loss [1, 2]. This condition affects nearly 1 in every 4,000 people worldwide [2, 3], and typically begins with night blindness, loss of peripheral vision, and progressive visual field loss, ultimately leading to the loss of central vision [4]. It impacts quality of life, leading to a loss of independence and a decline in psychological health [5]. Understanding the genetic and molecular basis of retinitis pigmentosa has been a significant focus of research, with limited therapeutic success, limited to visual rehabilitation and symptomatic management [6].

X-linked retinitis pigmentosa (XLRP) is one of the most severe and clinically complex forms of retinitis pigmentosa [7], accounting for 10–20% of all retinitis pigmentosa cases [8]. Pathogenic variants in the retinitis pigmentosa GTPase regulator (RPGR) gene, located at Xp11.4 [9], account for 70–80% of XLRP cases [10]. Its management typically includes dietary supplementation, low-vision assistive devices, and rehabilitation [7].

Using adeno-associated viral (AAV) vectors for gene therapy has become revolutionary for treating monogenic retinal diseases, including RPGR-associated XLRP [11, 12]. AAV vectors are non-pathogenic, exhibit minimal immunogenicity, and can achieve sustained transgene expression without integrating into the host genome [13]. AAV serotypes such as AAV5 and AAV8 demonstrate efficient transduction of retinal photoreceptors and retinal pigment epithelium cells, making them suitable vehicles for RPGR gene replacement [14]. Following subretinal injection, AAV vectors deliver functional copies of the RPGR gene to the photoreceptors, aiming to restore normal protein function, preserve photoreceptor structure, and maintain or improve visual function [15].

Early-phase clinical trials report improvements in visual function parameters, including best-corrected visual acuity and visual field sensitivity, and anatomical preservation on optical coherence tomography [16]. The favorable safety profile observed in initial cohorts and functional benefit has generated considerable optimism regarding the therapeutic potential of AAV RPGR gene therapy [17, 18]. Across studies, the outcome measures employed across studies have been diverse, encompassing structural endpoints (such as optical coherence tomography and fundus autofluorescence), functional assessments (including visual acuity, visual fields, microperimetry, and electroretinography), and patient-reported outcomes [19]. Additionally, safety concerns, including the potential for intraocular inflammation, inadvertent photoreceptor toxicity from surgical manipulation or immune responses, and the durability of therapeutic effect over extended follow-up periods, remain incompletely characterized [20, 21]. Therefore, this systematic review critically appraised the available evidence, synthesized functional and safety outcomes, and evaluated product-specific dose–safety patterns of AAV-mediated RPGR gene therapy for patients with XLRP. 

### Research purpose

 The purpose of this study was to critically appraise and quantitatively synthesize the efficacy and safety evidence for AAV-mediated RPGR gene therapy in XLRP.

## Methodology

### Protocol and registration

This project’s research protocol was formally registered with the International Prospective Register of Systematic Reviews (PROSPERO) under the registration number (CRD420261454510). This study was conducted according to the Preferred Reporting Items for Systematic Reviews and Meta-Analysis (PRISMA) statement [22]. As this study was based on previously published data and did not involve direct human or animal participants, Institutional Review Board approval and informed consent were not required.

### Data sources

A researcher (K. Y. C.) conducted an exhaustive search of Scopus, PubMed, Cochrane Library, ScienceDirect, and Google Scholar to identify peer-reviewed, original research from the inception of each database to July 11, 2026.

### Search strategy

A scoping search was conducted using Google Scholar and PubMed to evaluate the volume of available literature and identify relevant keywords. Key concepts related to AAV gene therapy and retinitis pigmentosa were used to build a comprehensive list of search terms and subject headings.

The keywords were combined using Boolean operators (AND/OR) to formulate specific search strings. The string was pilot-tested to confirm its reliability in retrieving relevant studies and was adjusted as needed. A research author (K. Y. C.) executed the final searches, and all search histories and outputs were documented in Microsoft Excel (2021) to maintain a transparent and replicable record.

### Database-specific search strategies

All databases were searched on July 11, 2026. The PubMed search was: ((“Retinitis Pigmentosa GTPase Regulator“[Mesh] OR RPGR[Title/Abstract] OR “retinitis pigmentosa GTPase regulator“[Title/Abstract]) AND (“Retinitis Pigmentosa, X-Linked“[Mesh] OR “X-linked retinitis pigmentosa“[Title/Abstract] OR XLRP[Title/Abstract]) AND (“Genetic Therapy“[Mesh] OR “gene therapy“[Title/Abstract] OR “gene augmentation“[Title/Abstract]) AND (“Adeno-Associated Virus“[Mesh] OR AAV[Title/Abstract] OR “adeno-associated virus“[Title/Abstract] OR “adeno-associated viral“[Title/Abstract])).

The Scopus search was: TITLE-ABS-KEY((RPGR OR “retinitis pigmentosa GTPase regulator”) AND (“X-linked retinitis pigmentosa” OR XLRP) AND (“gene therapy” OR “gene augmentation”) AND (AAV OR “adeno-associated virus” OR “adeno-associated viral”)).

The Cochrane Library search was: ([mh “Retinitis Pigmentosa, X-Linked”] OR “X-linked retinitis pigmentosa”:ti, ab, kw OR XLRP: ti, ab, kw) AND (RPGR: ti, ab, kw OR “retinitis pigmentosa GTPase regulator”:ti, ab, kw) AND ([mh “Genetic Therapy”] OR “gene therapy”:ti, ab, kw OR “gene augmentation”:ti, ab, kw) AND ([mh “Adeno-Associated Virus”] OR AAV: ti, ab, kw OR “adeno-associated virus”:ti, ab, kw).

The ScienceDirect search was: (“X-linked retinitis pigmentosa” OR XLRP) AND (RPGR OR “retinitis pigmentosa GTPase regulator”) AND (“gene therapy” OR “gene augmentation”) AND (AAV OR “adeno-associated virus”).

The Google Scholar search was: “X-linked retinitis pigmentosa” RPGR (“gene therapy” OR AAV OR “adeno-associated virus”). Results were ranked by relevance, the first 200 records were screened, and 23 topical records were exported before deduplication.

### Duplicate-removal procedure

Search outputs were exported with complete citation metadata and digital object identifiers into Zotero version 6.0.36. Exact duplicates were merged by digital object identifier, PubMed identifier, title, author, and publication year. Near-duplicate records were compared manually, retained under the most complete bibliographic record, and linked to all associated reports. Sixty-seven duplicate records were removed, and one retracted or automatically flagged record was confirmed by a reviewer before exclusion.

### Study selection and screening

The database search results were compiled in a Zotero library (v. 6.0.36). The software’s tools were used to identify and automatically merge duplicate entries. Additionally, any records flagged as retracted were manually removed. Two research authors (K. Y. C. and H. C. C.) independently screened the titles and abstracts of all non-duplicate records. The remaining records were retrieved for full-text evaluation against the predefined eligibility criteria. Any conflicts were settled by discussion or the intervention of a third author (C.M.C.) until a consensus was reached. All reasons for excluding articles at the full-text stage were carefully recorded and are presented in a PRISMA flow diagram [22].

### Eligibility criteria

This review was limited to original research articles published in peer-reviewed journals that explored the efficacy and safety of AAV-mediated gene therapy in retinitis pigmentosa. Modified PICOS criteria were used in the selection of eligible studies [23].

The PICOS framework was defined as follows:

- **Population (P)**: Human subjects with X-linked Retinitis Pigmentosa.
- **Intervention (I)**: Different dosages of AAV RPGR Gene Therapy.
- **Comparison (C)**: A comparison group, such as a placebo or other control, wherever available.
- **Outcomes (O)**: Visual acuity, retinal sensitivity, visual function, safety and tolerability.
- **Study Design (S)**: Clinical trials, experimental studies, and Randomized controlled studies.

### Exclusion criteria

Preclinical research studies were ineligible. Furthermore, the review excluded review articles, editorials, commentaries, book chapters, opinion pieces, and reports without extractable original human clinical outcome data. Publications in languages other than English or for which the full text could not be obtained were also excluded.

### Risk of bias assessment

The risk of bias evaluation for each included study was independently conducted by two research authors (K. Y. C. and H. C. C.) using the ROBINS-I and the ROB 2 [24]. The ROBINS-I tool assessed biases due to confounding variables, participant selection, intervention classification, deviations from the intended intervention, missing data, outcome measurement, and selective reporting of results [25]. On the other hand, for the two Randomized Controlled Trials (RCTs) included, ROB 2 evaluated potential bias in the randomization process, deviations from the intervention, missing outcome data, outcome measurement, and the selection of reported results [26]. Any conflicts were settled by discussion or the intervention of a third author (C. M. C.) until a consensus was reached.

### Data selection and extraction

A review author (K. Y. C.) extracted relevant information from each of the included studies. A second author (H. C. C.) then verified the extracted data for accuracy and consistency. Any conflicts were settled by discussion or the intervention of a third author (C. M. C.) until a consensus was reached. Key data points extracted included: study ID, study design, country, sample size, condition, intervention, outcome measures, and findings.

### Cohort linkage and overlapping populations

Publications were linked by trial registration, investigational product, recruitment period, dose cohort, sample size, and follow-up interval. Reports from XIRIUS, XOLARIS, MGT009, HORIZON, and DAWN were mapped at the cohort level. The report rather than the participant remained the descriptive unit in Table 1; Figs. 6, 7, 8, 9, 10, 11, 12, 13 and 14, while overlap-adjusted sensitivity analyses retained one report per trial programme, outcome, and follow-up interval to prevent repeated participant contribution.

### Standardized outcome definitions

Retinal sensitivity improvement comprised a prespecified responder definition or an increase in microperimetric or static-perimetric sensitivity within the analyzed retinal grid. Visual function improvement comprised best-corrected visual acuity, low-luminance visual acuity, validated microperimetry response, or a prespecified functional responder endpoint. Best-corrected visual acuity and low-luminance visual acuity were expressed in Early Treatment Diabetic Retinopathy Study letters. MAIA referred to Macular Integrity Assessment microperimetry. A 7-dB responder required an increase of at least 7 dB at five or more prespecified loci; a visual-acuity responder required a gain of at least 5 letters when this threshold was reported. Ellipsoid-zone recovery denoted increased area, width, reflectivity, or reappearance on optical coherence tomography. Safety outcomes were classified as treatment-related, surgery-related, corticosteroid-related, ocular, nonocular, serious, and nonserious.

### Handling missing data

In cases where essential data was missing, the research team contacted the study’s corresponding author via email to request clarification or additional information. When a response was not received after two attempts, the analysis proceeded using only the available data. When quantitative data required for meta-analysis were absent, estimates were calculated from other reported statistics. Any study with significant missing outcome data that could not be resolved was included in the qualitative summary but omitted from the meta-analysis.

### Data analysis

The qualitative results were thematically analyzed to identify common patterns, concepts, and themes related to the efficacy and safety of AAV RPGR gene therapy in retinitis pigmentosa [27]. Data were systematically coded and categorized under the following themes: visual acuity, retinal sensitivity, visual function, safety and tolerability.

### Meta-analysis

An author (K. Y. C.) used the Comprehensive Meta-analysis software for datasets with sufficient homogeneity [28]. This analysis utilized effect size data based on proportions and rates, with dichotomous data entered as event rates and sample sizes. The final results were presented in forest plots.

### Statistical specification and sensitivity analyses

For each outcome, the numerator represented participants meeting the reported endpoint and the denominator represented participants with evaluable data. Event proportions were transformed to the logit scale and pooled using inverse-variance fixed-effect models, matching the estimates displayed in Figs. 6, 7, 8, 9, 10, 11, 12, 13 and 14. A continuity correction of 0.5 was applied only to zero-event or all-event cells. Two-sided 95% confidence intervals were calculated on the logit scale and back-transformed. Cochran Q, I², and between-study variance τ² were reported; τ² was estimated using the DerSimonian-Laird method. Random-effects estimates and 95% prediction intervals were calculated as sensitivity analyses. Additional sensitivity analyses retained one report per trial programme and outcome and compared lower with higher vector exposure within each product-specific dosing system.

## Results

### Study selection and screening

**Fig. 1:**
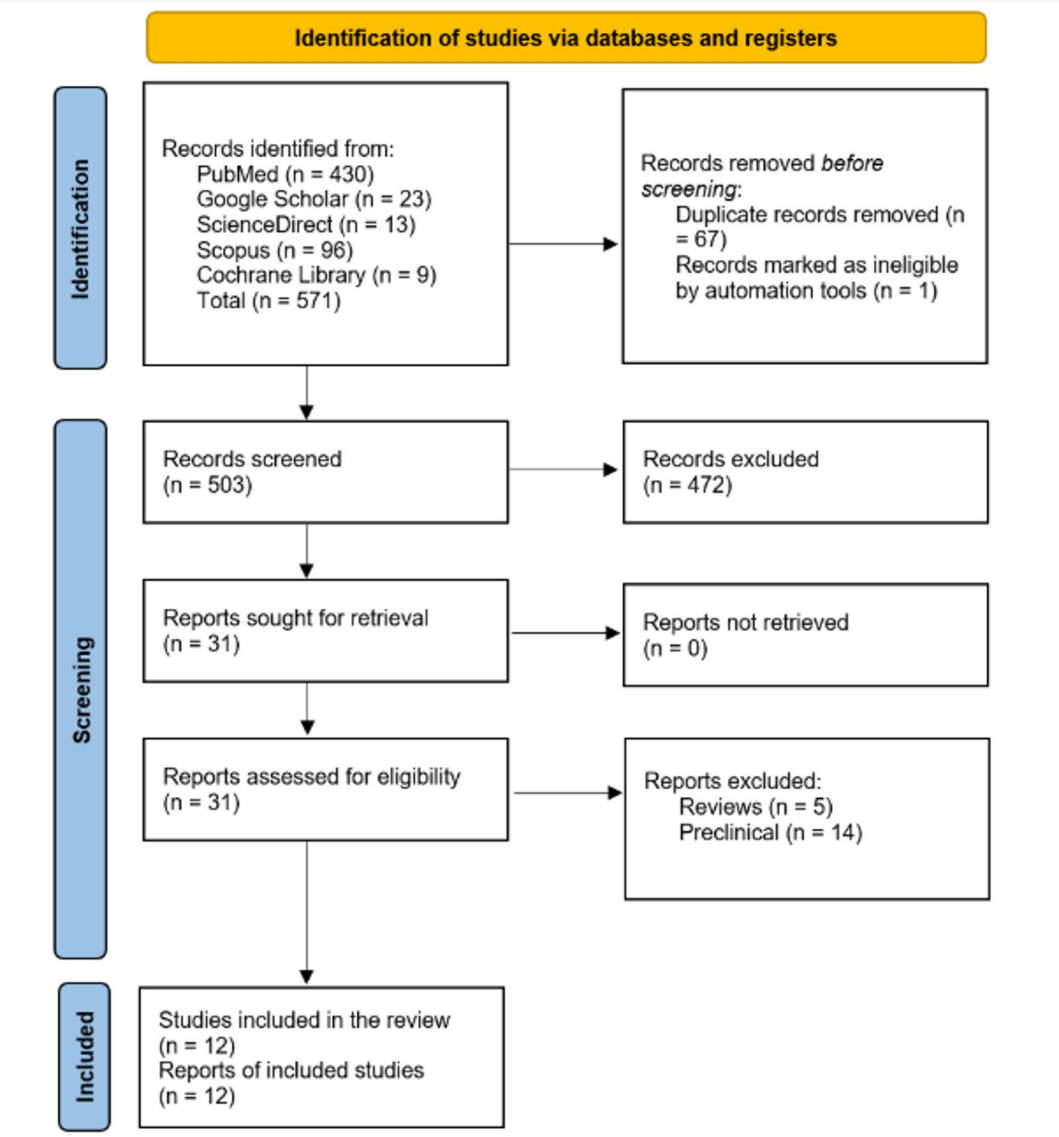
PRISMA flow diagram [22]

This PRISMA flow diagram outlines the systematic selection of studies included in the review. A total of 571 records were initially identified across five databases, comprising 430 from PubMed, 23 from Google Scholar, 13 from ScienceDirect, 96 from Scopus, and 9 from the Cochrane Library. After removing 67 duplicate records and one record deemed ineligible by automation tools, 503 unique studies remained for screening. Of these, 472 were excluded based on titles and abstracts, leaving 31 reports sought for full-text retrieval, all of which were successfully obtained. Following eligibility assessment, 19 reports were excluded—five being review articles and 14 classified as preclinical studies—resulting in the inclusion of 12 studies in the final systematic review (Fig. 1).

### Risk of bias assessment

**Fig. 2:**
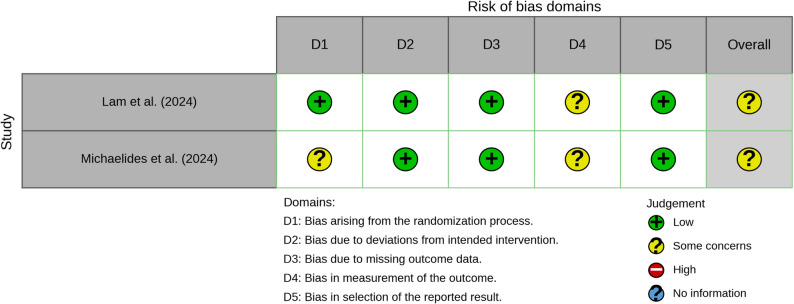
Traffic lights plot of the ROB 2.0 risk of bias assessment

Figure 2 presents the study-level Risk of Bias 2 assessment for the two randomized AAV-RPGR gene-therapy reports included in this review of X-linked retinitis pigmentosa. Lam et al. (2024) was judged low risk for deviations from intended interventions, missing outcome data, and selective reporting, with some concerns for randomization and outcome measurement. Michaelides et al. (2024) showed the same pattern; both studies therefore received an overall judgment of some concerns rather than high risk (Fig. 2).

**Fig. 3:**
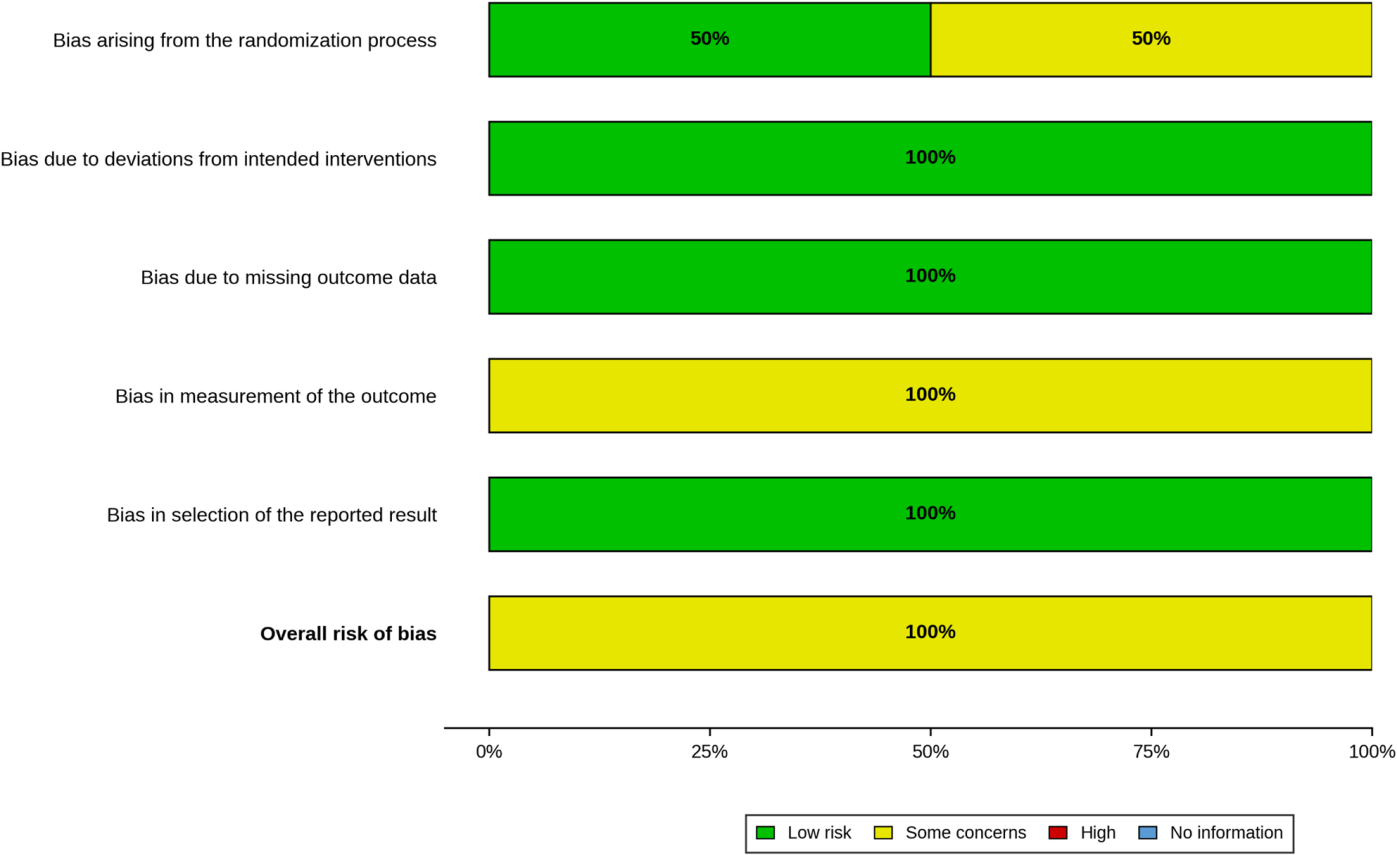
Summary plot of the ROB 2.0 risk of bias assessment

Figure 3 summarizes these randomized-trial judgments across domains. Deviations from intended interventions, missing outcome data, and selective reporting were low risk in 100% of studies. Randomization was low risk in 50% and raised some concerns in 50%, whereas outcome measurement raised some concerns in 100%. Accordingly, the overall Risk of Bias 2 judgment was some concerns for all randomized evidence (Fig. 3).

**Fig. 4:**
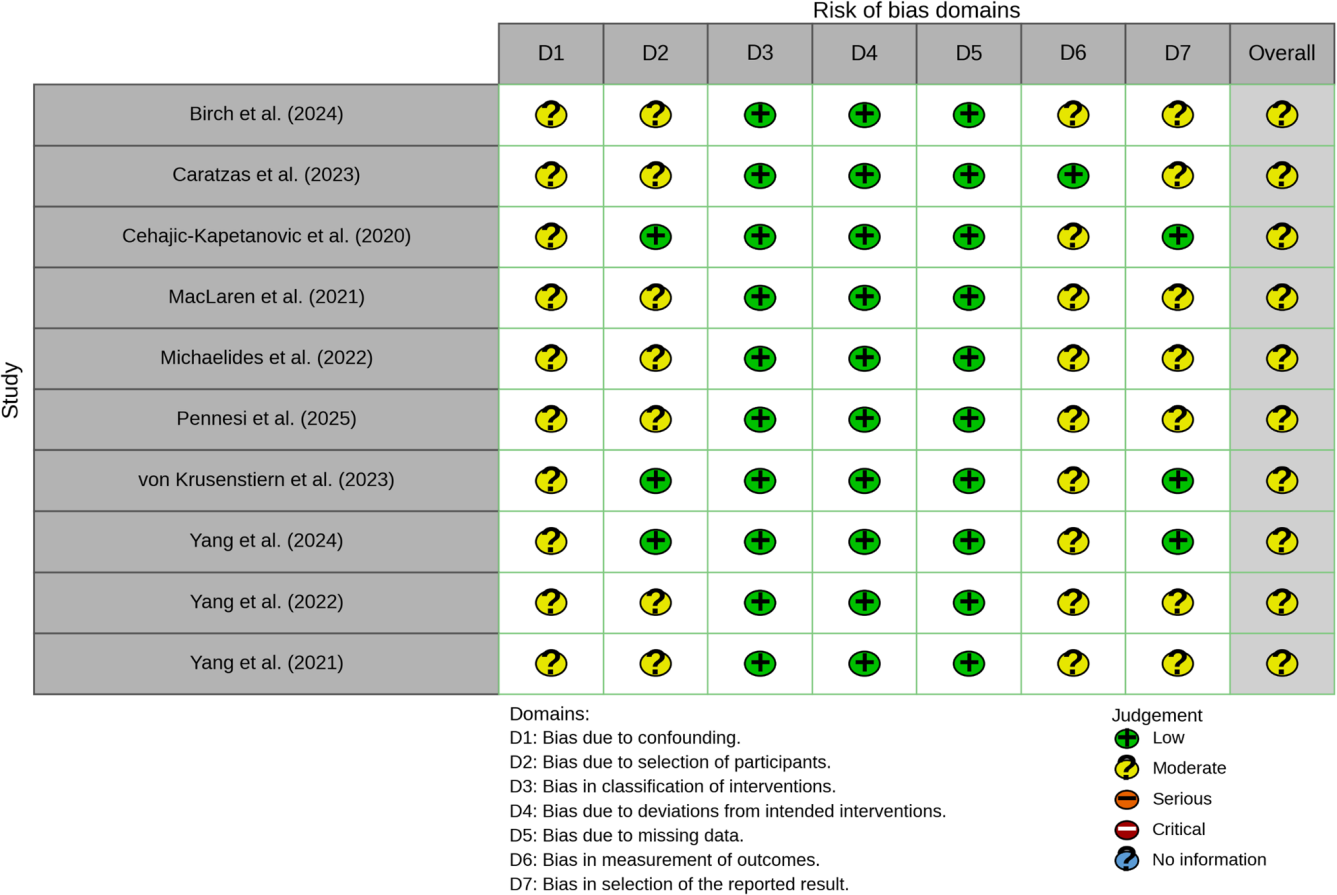
Traffic light plot of ROBINS-I risk of bias assessment

Figure 4 shows the ROBINS-I assessment for ten nonrandomized or secondary reports from the XIRIUS, MGT009, HORIZON, and DAWN programmes. Intervention classification, deviations from intended interventions, and missing data were consistently low risk. Moderate concerns were concentrated in confounding, participant selection, outcome measurement, and selective reporting, reflecting open-label dose-escalation designs, post hoc analyses, small cohorts, natural-history comparisons, and limited adjustment for prognostic differences. All reports received an overall moderate-risk judgment, with no serious or critical ratings (Fig. 4).

**Fig. 5:**
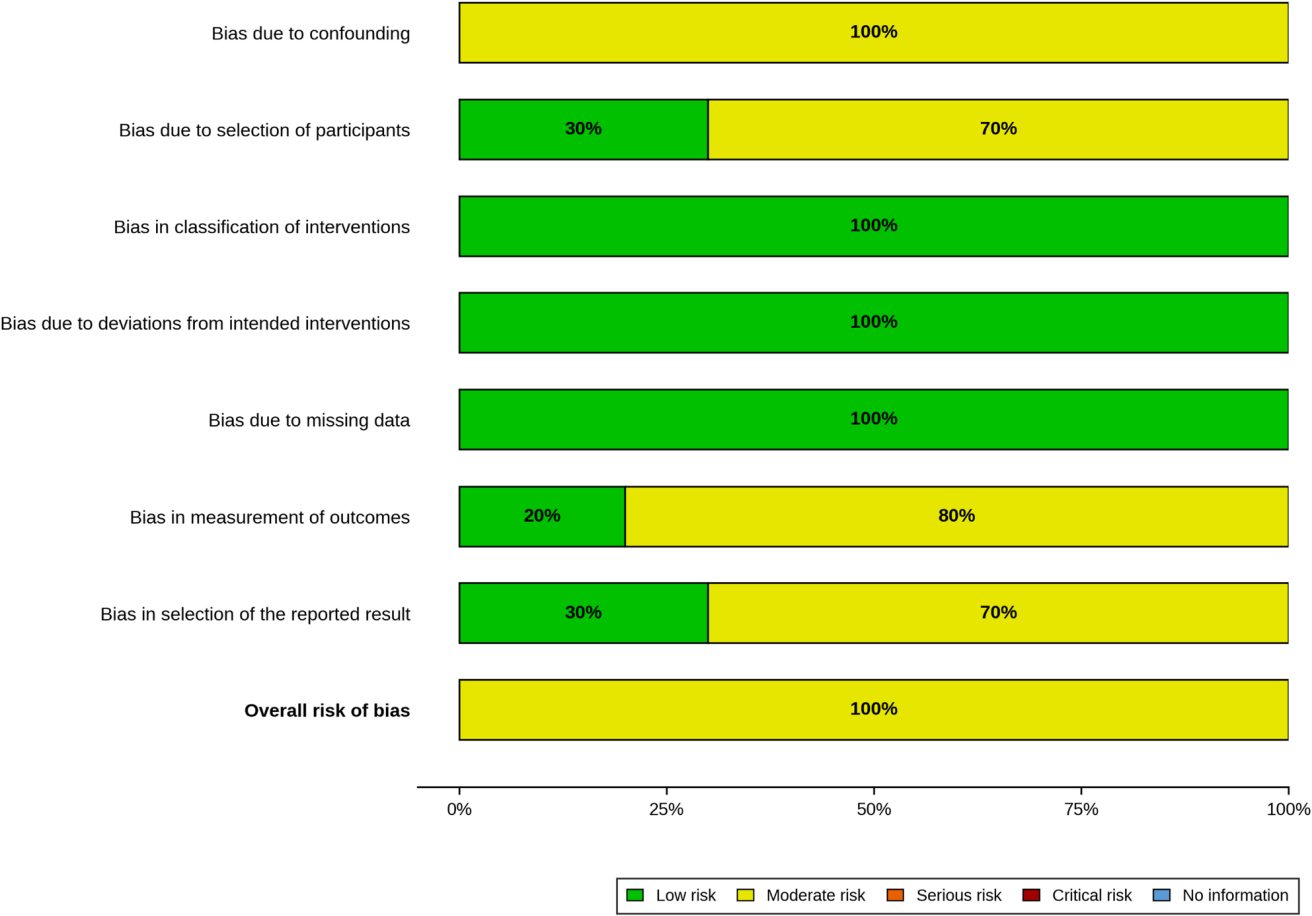
Summary plot of the ROBINS-I risk of bias assessment

Figure 5 quantifies this pattern. Confounding was moderate in 100% of reports; participant selection was low in 30% and moderate in 70%; outcome measurement was low in 20% and moderate in 80%; and selective reporting was low in 30% and moderate in 70%. Intervention classification, protocol deviations, and missing data were low risk in 100%. Overall, the evidence supports cautious confidence in the observed functional and safety signals, while emphasizing the need for masked assessment, prespecified analyses, and controlled phase 3 confirmation (Fig. 5).

### Overview of study characteristics

This analysis included 12 clinical reports examining AAV-RPGR gene therapy between 2020 and 2025. Sample sizes ranged from 2 to 103 participants across the XIRIUS/XOLARIS, MGT009, HORIZON, and DAWN evidence program. The evaluated products comprised cotoretigene toliparvovec, also reported as BIIB112 or AAV8-RPGR; botaretigene sparoparvovec, also reported as AAV5-hRKp.RPGR; and laruparetigene zovaparvovec, also reported as laru-zova or AGTC-501. All interventional products were delivered by subretinal injection, with product-specific vector concentration, total vector genomes per eye, injection volume, and dose cohort summarized in Table 1. Trial-programme linkage, overlapping reports, and overlap-adjusted analytical roles are summarized in Table 2, whereas product-specific dose-stratified safety findings are presented in Table 3.

**Table 1:** Study characteristics

Study ID	Study design	Country	Sample size	Condition	Intervention	Synthesized outcomes	Integrated findings
Birch et al. (2024) [29]	Phase 1/2 post hoc analysis	USA	29 enrolled; high-dose microperimetry subset	RPGR-associated XLRP	AGTC-501/laru-zova (rAAV2tYF-GRK1-RPGR), single subretinal administration; high-dose cohorts analyzed.	MAIA retinal sensitivity, responder distribution, ocular and systemic safety.	Microperimetry demonstrated localized sensitivity gains in treated retinal regions. Findings were linked to the HORIZON cohort and interpreted with the 24-month programme results.
Caratzas et al. (2023) [30]	Phase 1/2 secondary immunogenicity analysis	USA and UK	49 enrolled; 45 treated	RPGR-associated XLRP	Botaretigene sparoparvovec (AAV5-hRKp.RPGR); 1.0 × 10¹¹, 2.0 × 10¹¹, or 4.0 × 10¹¹ vector genomes/mL in 0.1–0.8 mL subretinal volumes.	Humoral immunogenicity, ocular inflammation, treatment-emergent adverse events.	Antibody status showed no consistent dose-related pattern. Ocular inflammation was concentrated in intermediate- and high-concentration cohorts and responded to corticosteroid treatment.
Cehajic-Kapetanovic et al. (2020) [31]	Phase 1/2 first-in-human dose-escalation trial	United Kingdom and USA	18 genetically confirmed male participants	RPGR-associated XLRP	AAV8.coRPGR/cotoretigene toliparvovec; 5 × 10⁹ to 4 × 10¹¹ genome particles/eye, corresponding to 5 × 10¹⁰ to 5 × 10¹² genome particles/mL.	Best-corrected visual acuity, retinal sensitivity, dose response, inflammation, ocular and nonocular adverse events.	Retinal sensitivity improved in selected treated eyes. Inflammation occurred in 0/9 participants across lower-dose cohorts and 7/9 across higher-dose cohorts; events were treated with corticosteroids.
Lam et al. (2024) [32]	Phase 2/3 randomized dose-expansion trial	United Kingdom and USA	29 total: 10 low dose, 10 high dose, 9 untreated control	RPGR-associated XLRP	Cotoretigene toliparvovec/BIIB112 (AAV8-RPGR); 5 × 10¹⁰ vector genomes/eye or 2.5 × 10¹¹ vector genomes/eye, single subretinal injection.	Microperimetry responder endpoint, mean retinal sensitivity, best-corrected and low-luminance visual acuity, ocular serious adverse events.	The primary responder endpoint showed comparable group proportions. Secondary mean-sensitivity and low-luminance visual-acuity measures favored the low-dose group; ocular serious adverse events occurred in 3/10 low-dose and 7/10 high-dose participants.
MacLaren et al. (2021) [33]	Phase 1/2 dose-expansion and natural-history comparison	United Kingdom and USA	18 treated participants; linked XOLARIS controls	RPGR-associated XLRP	BIIB112/cotoretigene toliparvovec (AAV8-RPGR), single subretinal administration across escalating exposure levels.	Retinal sensitivity, visual acuity, low-luminance visual acuity, inflammation, tolerability.	Functional responses appeared early and persisted through 12 months in selected treated eyes. Inflammatory events occurred more frequently at higher exposure and were managed with corticosteroids.
Michaelides et al. (2024) [17]	Phase 1/2 dose-escalation and randomized expansion trial	United States and Europe	49 enrolled; 45 treated	RPGR-associated XLRP	Botaretigene sparoparvovec (AAV5-hRKp.RPGR); adults received 1.0 × 10¹¹, 2.0 × 10¹¹, or 4.0 × 10¹¹ vector genomes/mL; pediatric participants received 2.0 × 10¹¹ vector genomes/mL.	Best-corrected and low-luminance visual acuity, retinal sensitivity, inflammation, ocular serious adverse events, intraocular pressure.	Functional gains were observed in selected dose groups. Twenty-five of 45 treated participants experienced ocular inflammation, 16/45 had increased intraocular pressure, and immediate-treatment serious ocular events included retinal detachment and uveitis.
Michaelides et al. (2022) [34]	Phase 1/2 localized perimetry analysis	United States and Europe	2 intensively characterized participants	RPGR-associated XLRP	Botaretigene sparoparvovec (AAV5-hRKp.RPGR), 0.3–0.8 mL subretinal administration.	Static perimetry within predefined visual-field regions, retinal sensitivity, photoreceptor function.	Localized improvements were detected within treated retinal regions, supporting spatial alignment of functional testing with the subretinal bleb.
Pennesi et al. (2025) [35]	Phase 2 DAWN preliminary analysis	USA	6 previously treated participants	RPGR-associated XLRP	Laruparetigene zovaparvovec/laru-zova (AGTC-501), 3.7 × 10¹¹ or 6.8 × 10¹¹ vector genomes/eye administered to the fellow eye.	Best-corrected visual acuity, retinal sensitivity, patient-reported outcomes, ocular and systemic safety.	Six-month observations supported bilateral functional assessment after sequential treatment and contributed visual-function responder data to the synthesis.
von Krusenstiern et al. (2023) [36]	XIRIUS post hoc analysis with XOLARIS comparison	United Kingdom and USA	18 XIRIUS treated; 103 XOLARIS comparators	RPGR-associated XLRP	Cotoretigene toliparvovec/BIIB112 (AAV8-RPGR), single subretinal treatment; natural-history comparison from XOLARIS.	Retinal sensitivity, best-corrected visual acuity, low-luminance visual acuity, treatment-emergent adverse events.	Retinal sensitivity improved in 12/18 treated participants in the report-level analysis. XOLARIS supplied an external natural-history context for longitudinal functional change.
Yang et al. (2024) [37]	Phase 1/2 HORIZON 24-month report	USA	29 treated participants	RPGR-associated XLRP	AGTC-501/laru-zova (rAAV2tYF-GRK1-RPGR), centrally or peripherally placed subretinal blebs across dose cohorts.	Macular sensitivity, best-corrected visual acuity, ellipsoid-zone appearance, grade ≥ 3 events, ocular serious adverse events, retinal detachment, intraocular pressure.	Functional and structural signals persisted through 24 months in selected eyes. All participants experienced at least one treatment-emergent adverse event; 11/29 had grade ≥ 3 events, 6/29 had treatment-related ocular serious events, and 4/29 developed retinal detachment.
Yang et al. (2022) [38]	Phase 1/2 HORIZON structural secondary analysis	USA	29 treated participants	RPGR-associated XLRP	AGTC-501/laru-zova, single subretinal administration with central and peripheral bleb placement.	Optical coherence tomography, ellipsoid-zone area and reflectivity, foveolar thickness, MAIA sensitivity.	Ellipsoid-zone recovery was reported in 9 eyes, with increased area or reflectivity in 6 eyes through months 12–18, alongside improved macular function.
Yang et al. (2021) [39]	Phase 1/2 HORIZON 6-month analysis	USA	29 treated participants	RPGR-associated XLRP	AGTC-501/laru-zova, centrally or peripherally targeted subretinal injection across escalating dose cohorts.	Macular sensitivity, ≥ 7-dB responder criterion, ≥ 5-letter visual-acuity gain, early safety.	Eighteen of 21 centrally treated participants achieved the ≥ 7-dB retinal-sensitivity criterion at five or more loci; visual-acuity and safety findings were subsequently extended in the 24-month report.

Abbreviations: AAV, adeno-associated virus; AE, adverse event; BCVA, best-corrected visual acuity; EZ, ellipsoid zone; gp, genome particles; IOP, intraocular pressure; LLVA, low-luminance visual acuity; MAIA, Macular Integrity Assessment; RCT, randomized controlled trial; RPGR, retinitis pigmentosa GTPase regulator; vg, vector genomes; XLRP, X-linked retinitis pigmentosa

**Table 2:** Cohort-level linkage, overlapping reports, and overlap-adjusted analytical role

Programme / registry	Product aliases	Core population	Reports in this review	Follow-up	Overlap structure	Sensitivity-analysis rule
XIRIUS NCT03116113	Cotoretigene toliparvovec; BIIB112; AAV8-RPGR; AAV8.coRPGR	18-participant dose escalation and 29-participant randomized expansion in genetically confirmed males	Cehajic-Kapetanovic 2020; MacLaren 2021; von Krusenstiern 2023; Lam 2024	Up to 12 months	Reports contain shared XIRIUS participants and complementary outcome definitions; XOLARIS provides untreated natural-history comparison.	Retain one XIRIUS report per outcome and follow-up; use the most complete denominator and prespecified endpoint.
XOLARIS NCT04926129	Prospective RPGR natural-history cohort	201 participants in the complete cohort; 103 used in the retinal-sensitivity comparison reported in this review	von Krusenstiern 2023 comparator data	24 months	Noninterventional cohort linked to XIRIUS analyses.	Use as external natural-history context; do not combine as an independent treated cohort.
MGT009 NCT03252847	Botaretigene sparoparvovec; AAV5-hRKp.RPGR	49 enrolled and 45 treated across adult, pediatric, and randomized expansion cohorts	Michaelides 2022; Caratzas 2023; Michaelides 2024	Up to 24 months	Secondary perimetry and immunogenicity reports share participants with the primary phase 1/2 report.	Retain Michaelides 2024 for overall efficacy and safety; use secondary reports only for unique spatial or immunogenicity outcomes.
HORIZON NCT03316560	AGTC-501; laru-zova; laruparetigene zovaparvovec; rAAV2tYF-GRK1-RPGR	29 treated participants across dose and bleb-location cohorts	Yang 2021; Yang 2022; Birch 2024; Yang 2024	6–24 months	All reports arise from the same HORIZON programme and report distinct time points or outcome domains.	Retain Yang 2024 for overall safety and 24-month efficacy; retain secondary reports only for unique microperimetry or structural endpoints.
DAWN NCT06275620	Laruparetigene zovaparvovec; laru-zova; AGTC-501	Previously treated males receiving fellow-eye treatment; preliminary report included 6 participants	Pennesi 2025	6 months in included report	Sequential fellow-eye treatment programme related to the AGTC-501 development pathway.	Analyze separately from HORIZON and retain as a distinct sequential-treatment cohort.
LUMEOS NCT04671433	Botaretigene sparoparvovec; AAV5-hRKp.RPGR	Phase 3 randomized programme; 105 participants registered	Contextualized in the Discussion; no report-level outcome entered in current pooled analyses	Primary completion in 2024	Later-phase programme following MGT009.	Use for clinical-development context after peer-reviewed outcome publication.

**Table 3:** Product-specific dose-stratified safety synthesis

Programme	Dose comparison	Safety endpoint	Lower exposure	Higher exposure	Effect estimate	Clinical synthesis
XIRIUS first-in-human	Cohorts 1–3 vs. cohorts 4–6	Intraocular inflammation	0/9 participants	7/9 participants	Risk ratio 15.00; 95% CI 0.98-228.91; 0.5 continuity correction	Inflammation clustered at higher AAV8.coRPGR exposure and was treated with corticosteroids.
XIRIUS randomized expansion	5 × 10¹⁰ vs. 2.5 × 10¹¹ vector genomes/eye	Ocular serious adverse events	3/10 participants	7/10 participants	Risk ratio 2.33; 95% CI 0.83–6.54	The lower-dose group combined favorable secondary functional signals with fewer ocular serious adverse events.
MGT009	1.0 × 10¹¹, 2.0 × 10¹¹, and 4.0 × 10¹¹ vector genomes/mL	Ocular inflammation and increased intraocular pressure	Events reported across cohorts with lower frequency at lower concentration	Inflammation included severe events in intermediate- and high-concentration cohorts	Descriptive concentration-stratified synthesis	Corticosteroid-responsive inflammation and pressure monitoring were integral to post-treatment care.
HORIZON	Escalating total vector genomes/eye and central vs. peripheral bleb placement	Grade ≥ 3 and ocular serious events	Lower and intermediate cohorts contributed the principal functional signals	3/4 participants at the highest dose developed retinal pigment epithelial changes	Descriptive product-specific synthesis	Dose selection integrated macular sensitivity, bleb location, retinal structure, and ocular safety.

### Cohort-linked and dose-stratified sensitivity analyses

Cohort linkage identified repeated reporting within XIRIUS, MGT009, and HORIZON. The overlap-adjusted visual-function analysis retained one report per independent programme and yielded 53.1% improvement (95% confidence interval, 36.1%-69.4%; I²=0%), closely matching the report-level estimate of 52.3%. Retinal-sensitivity reports were summarized at the programme level after overlap adjustment.

### Systematic review results

#### Visual acuity

Best-corrected visual acuity (BCVA) remained stable at 6 months, with a mean change of -0.1 letters (95% CI: -2.8 to + 2.6) in treated eyes, while untreated eyes showed a change of + 0.8 letters (95% CI: -1.3 to + 2.9) compared to baseline [31]. A separate controlled analysis reported a least squares mean change from baseline of 0.59 (95% CI: -1.19 to 2.37) for treatment participants and − 3.05 (95% CI: -5.58 to -0.52) for the control group [17]. Similarly, there were improvements in low-luminance visual acuity (LLVA), assessed by a gain of ≥ 15 Early Treatment Diabetic Retinopathy Study (ETDRS) letters in 3/11 (27%) XIRIUS-treated eyes, in as early as Month 1 and through 12 months of follow-up [33]. In another study, the mean change from baseline in microperimetry sensitivity improved significantly in the low-dose group compared to the control group at month 12 (*P* = 0.0350). In addition, a significant improvement in LLVA was observed in the low-dose group compared to the control group at month 12 (33.3% difference [80% CI, 14.7%–55.2%]; *P* = 0.0498) [32].

The mean change from baseline in LLVA in high dose group study eyes was + 10.6 letters at M3 (SD = 9.40, *n* = 7) and + 13.5 letters at M6 (SD = 10.31, *n* = 6), while the contralateral eyes had a mean change of + 1.6 letters at M3 (SD = 4.89, *n* = 7) and + 2.7 letters at M6 (SD = 6.56, *n* = 6) [17].

 Several reports described visual-acuity or low-luminance visual-acuity gains or responder outcomes in selected treated eyes [17, 33, 35, 37, 39]. Participants met a retinal-sensitivity responder criterion defined as an improvement of at least 7 dB from baseline at five or more prespecified central loci [36]. In addition, at 24 months, 55% (11/20) of centrally treated eyes gained ≥ 5 ETDRS letters versus 50% (4/8) of peripherally treated eyes. Corresponding fellow eyes showed 60% (12/20) and 37.5% (3/8) with any letter gains, respectively [37].

#### Retinal sensitivity

Participants showed improvements in retinal sensitivity [17, 36]. An increase in retinal sensitivity was observed in all three low-dose patients, improving from 0.5 dB to 3.4 dB at 1 month and to 6.6 dB at 3 months [31]. Similarly, in treated eyes, there was an improvement in mean MAIA sensitivity at 12 months [29]. In another study, the mean change from baseline in microperimetry sensitivity improved significantly in the low-dose group compared to the control group at month 12 [32]. Moreover, at 12 months, baseline-subtracted retinal sensitivity within V30 improved by 1.94 dB-sr in one patient and 0.64 dB-sr in another, with corresponding within-bleb increases of 0.66 and 0.21 dB-sr [34]. At 6 and 12 months, microperimetry revealed improved retinal sensitivity in the treated eye alongside progressive retinal pigment epithelium (RPE) depigmentation and structural changes at and around the injection site [37]. Mean retinal sensitivity also showed improvements, indicating enhanced rod-mediated function under dark-adapted conditions, with the least squares mean change from baseline at Week 26 of 0.88 dB (95% CI: 0.35 to 1.41) [17]. There were improvements in photoreceptor function [34].

### Visual function

Treatment with BIIB112 resulted in early and durable improvements in visual function [33]. Following subretinal macular injection, the ellipsoid zone (EZ) recovered in 9 eyes (69%), with 6 showing increased EZ area and reflectivity by month 12, sustained to month 18. Treated eyes demonstrated significant improvement in macular function on Macular Integrity Assessment (MAIA) microperimetry (*p* = 0.003) and stable foveolar thickness on optical coherence tomography (OCT) (*p* = 0.0003) [38]. In the centrally dosed group, 18 of 21 participants demonstrated a ≥ 7 dB improvement in at least five loci within the central macula (*p* ≤ 0.05). Additionally, 11 of 20 participants showed a ≥ 5-letter BCVA gain in treated eyes compared with fellow eyes (*p* = 0.001), while peripherally treated eyes maintained stable BCVA [39]. Similarly, in treated eyes, there was an improvement in mean MAIA sensitivity at 12 months [29]. At week 26, the least-squares mean change from baseline in BCVA was 0.59 Early Treatment Diabetic Retinopathy Study (ETDRS) letters (95% CI, −1.19 to 2.37) [17].

### Safety and tolerability

Across the botaretigene sparoparvovec reports, most adverse events were mild to moderate or managed with corticosteroid therapy, and ocular serious adverse events were also reported [17, 29, 30, 32, 34, 35, 37, 38]. Subretinal administration of AGTC-501 produced functional signals across the evaluated doses with product- and dose-specific ocular safety findings [39]. Mild retinal inflammation occurred in 7 of 9 participants in the higher-dose cohorts and resolved with corticosteroid treatment [31]. In another study, 55.6% of participants reported mild ocular inflammation, while one participant in the intermediate dose group and one in the high dose group had severe ocular inflammation [17]. Moreover, a study reported ocular inflammation in 12 participants [36], with twenty-five out of 45 participants experiencing mild to moderate ocular inflammation [17]. In addition, inflammation, primarily at higher doses, was successfully treated with oral corticosteroids [33].

No serious adverse events or dose-limiting toxicities were reported in the first 6 months. Eighteen participants reported 35 mild ocular and 20 non-ocular cases [31]. On the other hand, no dose-limiting events were reported [30, 33, 36, 38, 39]. Surgical-related adverse events and retinal detachment were observed [17]. In another study, patients reported non-serious adverse events related to the study agent [29]. Fewer serious adverse events occurred with low-dose compared with high-dose cotoretigene toliparvovec [32].

Patients experienced mild to moderate treatment-emergent adverse events, including increased intraocular pressure (IOP; 42%) and noninfectious retinitis (42%). Serious treatment-emergent adverse events, including reduced visual acuity and noninfectious retinitis, occurred in 3 participants (25%) [36]. In the 24-month HORIZON report, all 29 participants experienced at least one treatment-emergent adverse event [37]. In addition, increased IOP occurred in 16 of 45 participants (35.6%), with two cases attributed to treatment and four to surgery [17]. In addition, steroid-associated increases in IOP were observed in 5 participants (24%), including four who were centrally treated (19%) and one who was peripherally treated (13%). Moreover, one patient developed glaucoma, partially attributed to corticosteroid exposure, necessitating Ahmed valve implantation [37].

### Meta-analysis results

#### Retinal sensitivity improvement

**Fig. 6:**
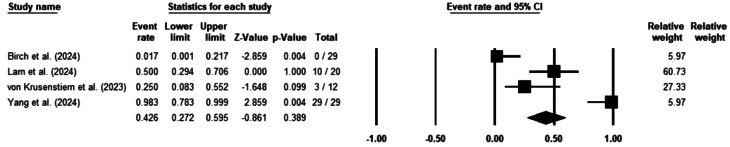
Forest plot of proportion meta-analysis of patients reporting improvement in retinal sensitivity [17, 28]

Figure 6 presents the forest plot for retinal sensitivity improvement. Three reports contributed 25 responders among 33 evaluable participants, producing a pooled proportion of 73.8% (95% confidence interval, 56.0%-86.1%; *p* = 0.011). Between-report heterogeneity was low (Q = 1.38, I²=0%, τ²=0), the random-effects sensitivity estimate was 73.8%, and the 95% prediction interval was 33.0%-94.1%.

**Fig. 7:**
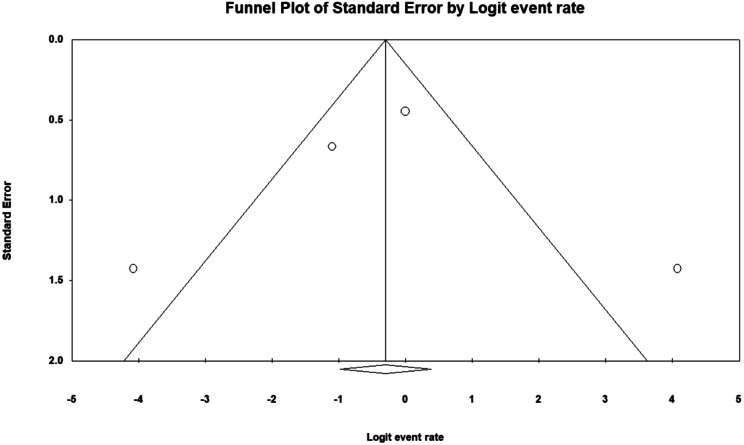
Funnel plot assessing publication bias in retinal sensitivity improvement following AAV-RPGR gene therapy for retinitis pigmentosa [33, 36]

Figure 7 presents the corresponding funnel plot for retinal sensitivity. The three-report configuration provides a descriptive visual assessment of small-study distribution and complements the cohort-linked sensitivity analysis.

### Visual function improvement

**Fig. 8:**
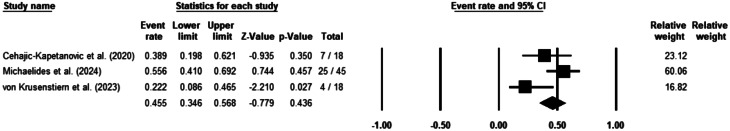
Forest plot of proportion meta-analysis of patients reporting improvement in Visual function [31, 33, 36, 39]

Figure 8 presents the forest plot for visual function improvement. Five reports contributed 28 responders among 52 evaluable participants, producing a pooled proportion of 52.3% (95% confidence interval, 38.0%-66.2%; *p* = 0.757). Heterogeneity was Q = 6.02, I²=33.5%, and τ²=0.253. The random-effects sensitivity estimate was 53.7% (95% confidence interval, 34.8%-71.5%), with a 95% prediction interval of 16.4%-87.3%.

**Fig. 9:**
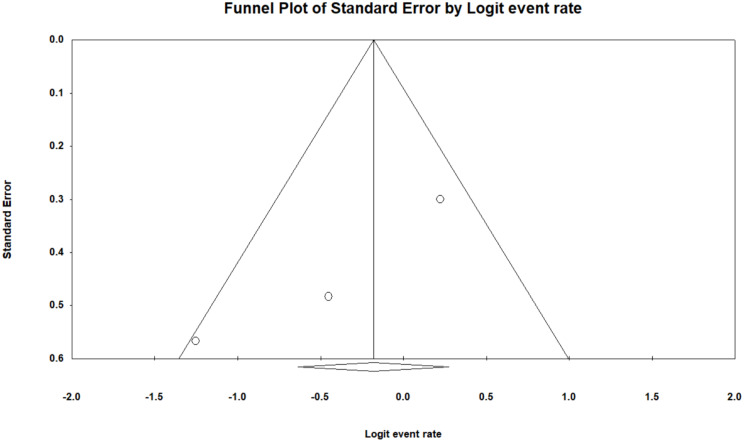
Funnel plot of proportion meta-analysis of patients reporting improvement in visual function [31, 33, 36, 39]

Figure 9 presents the corresponding funnel plot for visual function. The plot is interpreted together with the overlap-adjusted sensitivity estimate of 53.1%, which retained one report from each independent interventional programme contributing compatible data.

### Adverse events

**Fig. 10:**
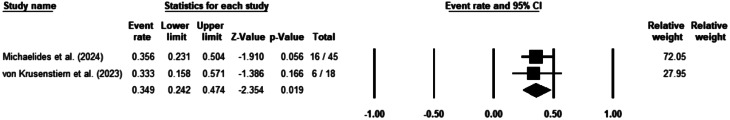
Forest plot of proportion meta-analysis of the occurrence of adverse events [29, 32, 36, 37]

Figure 10 presents the forest plot for report-defined adverse-event endpoints. Four reports contributed 42 participants with at least one reported adverse event among 90 evaluable participants, producing a pooled proportion of 42.6% (95% confidence interval, 27.2%-59.5%; *p* = 0.389). Heterogeneity was Q = 18.32, I²=83.6%, and τ²=3.388. The random-effects sensitivity estimate was 42.2% (95% confidence interval, 8.5%-85.1%), with a 95% prediction interval of 0.1%-99.8%.

**Fig. 11:**
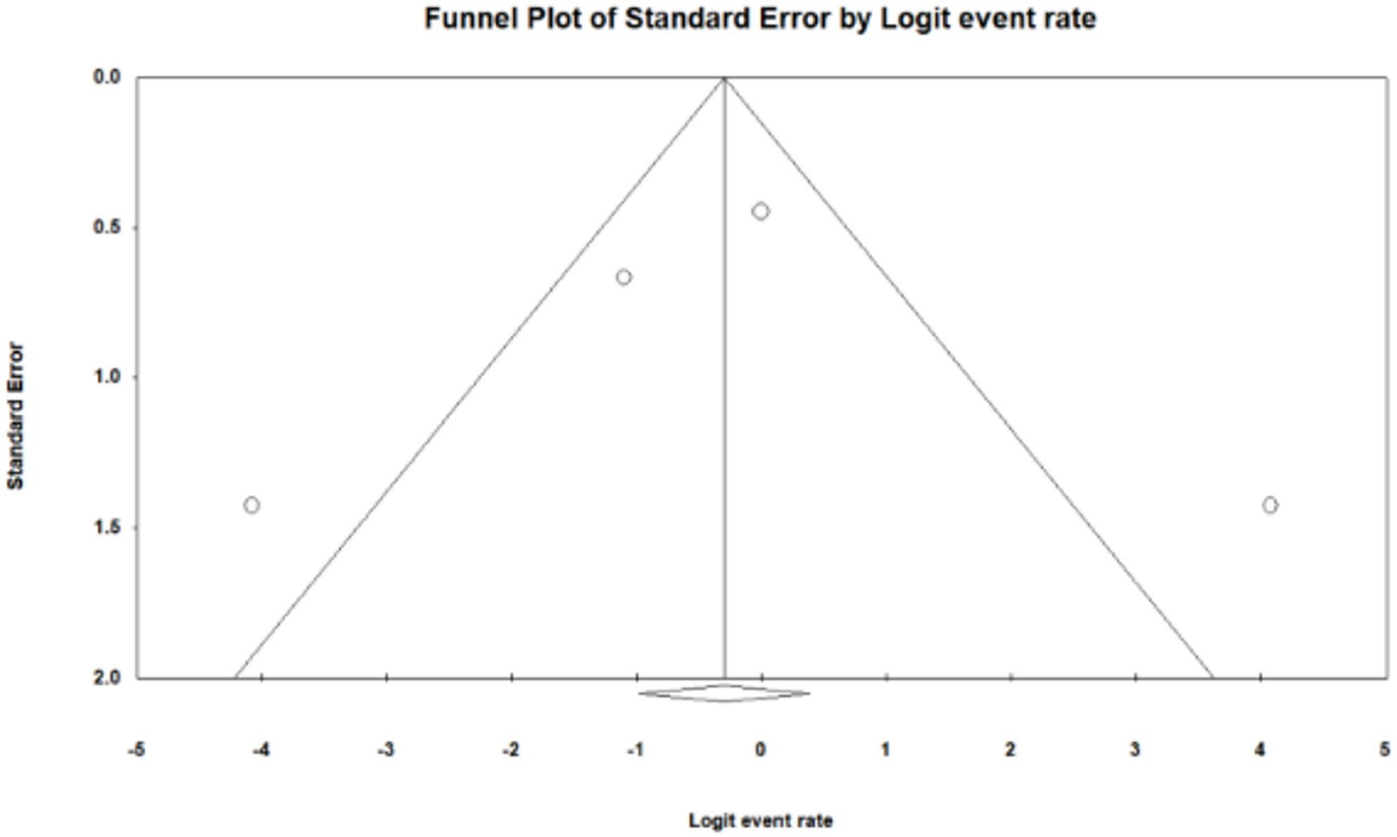
Funnel plot of proportion meta-analysis of the occurrence of adverse events [29, 32, 36, 37]

Figure 11 presents the corresponding adverse-event funnel plot. Event attribution was subsequently separated into treatment-related, surgery-related, corticosteroid-related, ocular, nonocular, serious, and nonserious categories in the structured safety synthesis.

### Intraocular inflammation

**Fig. 12:**
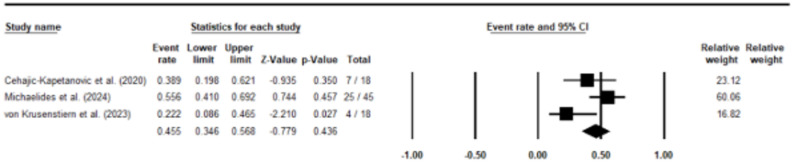
Forest plot of proportion meta-analysis of the occurrence of intraocular inflammation [17, 31, 36]

Figure 12 presents the forest plot for intraocular inflammation. Three reports contributed 36 events among 81 evaluable participants, producing a pooled proportion of 45.5% (95% confidence interval, 34.6%-56.8%; *p* = 0.436). Heterogeneity was Q = 5.70, I²=64.9%, and τ²=0.359. The random-effects sensitivity estimate was 40.3% (95% confidence interval, 22.5%-61.1%), with a 95% prediction interval of 2.7%-94.2%.

**Fig. 13:**
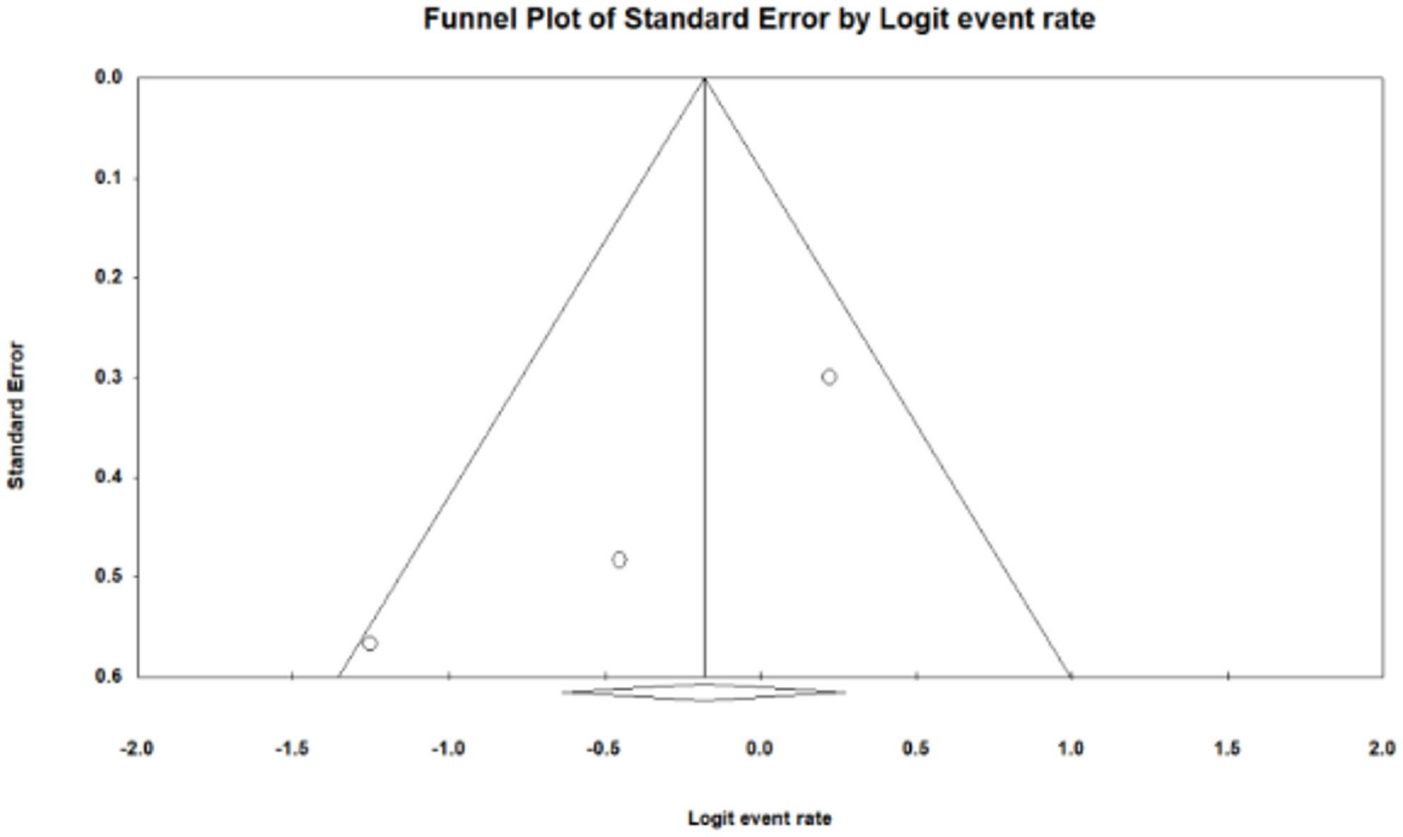
Funnel plot of proportion meta-analysis of the occurrence of intraocular inflammation [17, 31, 36]

Figure 13 presents the corresponding funnel plot for intraocular inflammation. Product-specific dose stratification demonstrated concentration-dependent clustering of inflammation, and all reported management pathways incorporated topical, periocular, or systemic corticosteroid therapy according to severity.

### IOP increase

**Fig. 14:**
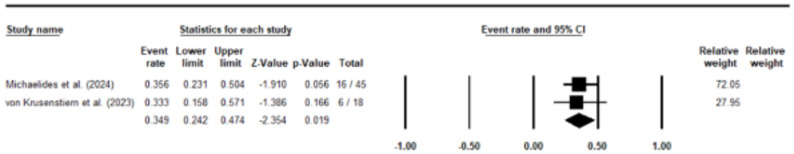
Forest plot of proportion meta-analysis of the occurrence of IOP increase [17, 36]

Figure 14 presents the forest plot for intraocular-pressure elevation. Two reports contributed 22 events among 63 evaluable participants, producing a pooled proportion of 34.9% (95% confidence interval, 24.2%-47.4%; *p* = 0.019). Heterogeneity was Q = 0.03, I²=0%, and τ²=0. The random-effects estimate was 34.9%, and the 95% prediction interval was 1.8%-93.9%. Reported management included corticosteroid adjustment, topical pressure-lowering medication, longitudinal pressure surveillance, and Ahmed valve implantation in one participant with glaucoma.

## Discussion

This study investigated the therapeutic efficacy and safety of AAV-RPGR gene therapy for X-linked retinitis pigmentosa. A meta-analysis demonstrated a pooled retinal sensitivity improvement proportion of 73.8% (95% confidence interval, 56.0%-86.1%; *p* = 0.011), representing a substantial clinical benefit in a condition characterized by progressive photoreceptor degeneration [33, 36]. Visual function improvement reached 52.3%, and the pooled-proportion test (*p* = 0.757) was accompanied by I²=33.5%, reflecting moderate variation across vector design, dosing strategy, outcome definition, and baseline disease stage [31, 33, 36, 39].

The observed visual acuity improvements, with treated eyes gaining + 10.6 to + 13.5 letters, compared to minimal changes in contralateral controls, highlight the treatment efficacy beyond natural disease fluctuations [17]. The temporal pattern of benefit, emerging as early as one month post-injection and sustained through 12–18 months, suggests successful transgene expression and functional photoreceptor rescue [29]. Moreover, the recovery of ellipsoid zone integrity in 69% of treated eyes further corroborates structural restoration accompanying functional gains [38].

The safety profile showed manageable adverse events, with intraocular inflammation (45.5%) and increased intraocular pressure (34.9%, *p* =0.019) [17, 36]. Most inflammatory responses were mild to moderate and responsive to corticosteroid therapy, consistent with anticipated immune responses to subretinal AAV administration. In addition, the dose-dependent increase in inflammation rates suggests that immune-mediated reactions occur with higher vector loads, highlighting the importance of dose optimization. No dose-limiting toxicity was reported in the included early-phase reports across the three principal product platforms: cotoretigene toliparvovec (BIIB112/AAV8-RPGR), botaretigene sparoparvovec (AAV5-hRKp.RPGR), and laruparetigene zovaparvovec (AGTC-501/laru-zova). In parallel, long-term surveillance is essential given the chronic nature of corticosteroid-responsive inflammation.

The dose-stratified synthesis provides a clinically actionable interpretation of ocular safety. In XIRIUS, intraocular inflammation occurred in 0/9 participants across lower-dose cohorts and 7/9 across higher-dose cohorts. In the randomized expansion, three ocular-related serious adverse events occurred in the low-dose group receiving 5 × 10¹⁰ vector genomes/eye, and seven occurred in the high-dose group receiving 2.5 × 10¹¹ vector genomes/eye. Across MGT009 and HORIZON, dose selection incorporated inflammatory grade, intraocular-pressure response, retinal pigment epithelial integrity, retinal detachment, and functional gain. These findings support product-specific dose optimization rather than direct conversion across vector genomes/mL, genome particles/mL, and vector genomes/eye.

### Novelty in relation to existing evidence

The 2026 Translational Vision Science & Technology RPGR meta-analysis synthesized five articles from three studies using comparative and continuous outcomes. The present review extends that evidence architecture by integrating 12 clinical reports, mapping XIRIUS, XOLARIS, MGT009, HORIZON, DAWN, and LUMEOS at the cohort level, retaining follow-up through 24 months, standardizing product aliases and outcome definitions, separating treatment-, surgery-, and corticosteroid-related events, and quantifying product-specific dose-safety patterns. The synthesis also links the foundational Cehajic-Kapetanovic trial, the Michaelides botaretigene programme, the randomized Lam XIRIUS analysis, the Yang HORIZON series, and later-phase LUMEOS development within one clinically structured framework. The mechanism underlying therapeutic benefit likely involves successful RPGR protein expression at the photoreceptor connecting cilium, restoring protein trafficking and preventing photoreceptor apoptosis [40, 41]. The superior outcomes in centrally treated versus peripherally treated eyes underscore the importance of targeting areas with preserved photoreceptor populations, suggesting therapeutic windows before irreversible degeneration occurs.

### Study strengths and global applicability

This analysis used objective outcome measures, including microperimetry and optical coherence tomography, minimizing subjective bias in efficacy assessment. These quantitative tools provide reproducible and standardized measurements of retinal structure and function, enabling reliable comparisons across studies and robust meta-analytical synthesis. In addition, the inclusion of contralateral untreated eyes as internal controls in multiple studies provides powerful within-patient comparisons, effectively controlling for inter-individual variability and disease progression. Moreover, the evaluation of multiple independent AAV vectors (AGTC-501, AAV5-RPGR, AAV8-RPGR, laru-zova) across different serotypes strengthens the generalizability of findings and suggests class-wide therapeutic effects rather than vector-specific artifacts. Furthermore, the extended follow-up periods, reaching 24 months, provide meaningful insights into the durability of therapeutic benefit and safety profile beyond the immediate post-operative effects, addressing critical clinical questions regarding sustained efficacy in this progressive condition.

The evidence was generated predominantly in the United States and United Kingdom, creating a clear agenda for broader multinational implementation research. Regional genetic architecture, RPGR variant distribution, baseline disease stage, age at treatment, retinal structure, surgical infrastructure, and access to corticosteroid and intraocular-pressure surveillance can influence observed outcomes. Trials and registries in Asia, Africa, Latin America, the Middle East, and other underrepresented regions can prospectively harmonize microperimetry, visual-acuity, optical coherence tomography, inflammation grading, corticosteroid protocols, and surgical-event reporting. This approach will strengthen population-specific treatment selection and support equitable access to post-treatment monitoring.

### Study implications and future research directions

These findings support continued clinical development of AAV-RPGR gene therapy for patients with confirmed RPGR variants and residual photoreceptor function. Patient selection can integrate age, phenotype, baseline retinal sensitivity, ellipsoid-zone preservation, bleb location, and vector-specific dose. Standardized surveillance pathways should include inflammation grading, corticosteroid exposure, intraocular-pressure measurement, retinal structural imaging, and prompt management of procedure-related events across treatment centers.

Head-to-head and platform-specific trials can refine vector selection, total dose, concentration, injection volume, and bleb placement. Long-term observational studies extending beyond five years should assess durability, anatomical preservation, patient-reported function, and late ocular events. Predictive biomarkers can enable response stratification and personalized dosing. Pediatric and globally diverse populations warrant dedicated evaluation, as earlier treatment can preserve a larger population of viable photoreceptors.

## Conclusions

This study demonstrates that AAV-RPGR gene therapy represents a clinically meaningful advancement in the treatment pathway for X-linked retinitis pigmentosa. Across 12 clinical reports, retinal sensitivity improvement reached 73.8%, visual function improvement reached 52.3%, intraocular inflammation reached 45.5%, and intraocular-pressure elevation reached 34.9%. Functional signals emerged as early as one month and persisted through 24 months in selected cohorts. A structured safety framework that separates vector-, surgery-, and corticosteroid-related events supports precise surveillance and product-specific dose selection.

Cohort-level linkage clarifies the relationships among XIRIUS, XOLARIS, MGT009, HORIZON, DAWN, and LUMEOS and enables overlap-adjusted interpretation. Standardized retinal-sensitivity, visual-acuity, microperimetry, optical coherence tomography, inflammation, and intraocular-pressure definitions provide a reproducible foundation for future evidence synthesis. Multinational trials, harmonized outcome reporting, long-term follow-up, and biomarker-guided selection will further define durable benefit and individualized treatment protocols.

## Data Availability

All data generated or analyzed during this study are included in this published article and its supplementary information files.
